# Prodromal Intestinal Events in Alzheimer’s Disease (AD): Colonic Dysmotility and Inflammation Are Associated with Enteric AD-Related Protein Deposition

**DOI:** 10.3390/ijms21103523

**Published:** 2020-05-15

**Authors:** Carolina Pellegrini, Simona Daniele, Luca Antonioli, Laura Benvenuti, Vanessa D’Antongiovanni, Rebecca Piccarducci, Deborah Pietrobono, Valentina Citi, Eugenia Piragine, Lorenzo Flori, Chiara Ippolito, Cristina Segnani, Pablo Palazon-Riquelme, Gloria Lopez-Castejon, Alma Martelli, Rocchina Colucci, Nunzia Bernardini, Maria Letizia Trincavelli, Vincenzo Calderone, Claudia Martini, Corrado Blandizzi, Matteo Fornai

**Affiliations:** 1 Department of Pharmacy, University of Pisa, 56126 Pisa, Italy; carolina.pellegrini@unipi.it (C.P.); simona.daniele@unipi.it (S.D.); rebecca.piccarducci@farm.unipi.it (R.P.); deborah.pietrobono@farm.unipi.it (D.P.); valentina.citi@unipi.it (V.C.); eugenia.piragine@farm.unipi.it (E.P.); lorenzo.flori@phd.unipi.it (L.F.); alma.martelli@unipi.it (A.M.); maria.trincavelli@unipi.it (M.L.T.); vincenzo.calderone@unipi.it (V.C.); 2 Unit of Pharmacology and Pharmacovigilance, Department of Clinical and Experimental Medicine, University of Pisa, 56126 Pisa, Italy; luca.antonioli@unipi.it (L.A.); laura.benvenuti@phd.unipi.it (L.B.); v.dantongiovanni@gmail.com (V.D.); matteo.fornai@unipi.it (M.F.); 3 Unit of Histology and Medical Embryology, Department of Clinical and Experimental Medicine, University of Pisa, 56126 Pisa, Italy; chiara.ippolito@unipi.it (C.I.); cristina.segnani@unipi.it (C.S.); nunzia.bernardini@med.unipi.it (N.B.); 4 Manchester Collaborative Centre for Inflammation Research, University of Manchester, Manchester M13 9PL, UK; pablopalazonriquelme@gmail.com (P.P.-R.); gloria.lopez-castejon@manchester.ac.uk (G.L.-C.); 5 Department of Pharmaceutical and Pharmacological Sciences, University of Padova, 35131 Padova, Italy; rok.colucci@gmail.com; 6 Interdepartmental Research Centre “Nutraceuticals and Food for Health”, University of Pisa, 56126 Pisa, Italy

**Keywords:** Alzheimer’s disease, mild cognitive impairment, colonic motility, enteric inflammation, NLRP3 inflammasome, interleukin-1β, β-amyloid protein, tau protein, α-synuclein, enteric neuronal coding, mitochondrial function

## Abstract

Increasing evidence suggests that intestinal dysfunctions may represent early events in Alzheimer’s disease and contribute to brain pathology. This study examined the relationship between onset of cognitive impairment and colonic dysfunctions in a spontaneous AD model before the full development of brain pathology. SAMP8 mice underwent Morris water maze and assessment of faecal output at four, six and eight months of age. In vitro colonic motility was examined. Faecal and colonic Aβ, tau proteins, α-synuclein and IL-1β were assessed by ELISA. Colonic citrate synthase activity was assessed by spectrophotometry. Colonic NLRP3, caspase-1 and ASC expression were evaluated by Western blotting. Colonic eosinophil density and claudin-1 expression were evaluated by immunohistochemistry. The effect of Aβ on NLRP3 signalling and mitochondrial function was tested in cultured cells. Cognitive impairment and decreased faecal output occurred in SAMP8 mice from six months. When compared with SAMR1, SAMP8 animals displayed: (1) impaired in vitro colonic contractions; (2) increased enteric AD-related proteins, IL-1β, active-caspase-1 expression and eosinophil density; and (3) decreased citrate synthase activity and claudin-1 expression. In THP-1 cells, Aβ promoted IL-1β release, which was abrogated upon incubation with caspase-1 inhibitor or in ASC^-/-^ cells. Aβ decreased mitochondrial function in THP-1 cells. In SAMP8, enteric AD-related proteins deposition, inflammation and impaired colonic excitatory neurotransmission, occurring before the full brain pathology development, could contribute to bowel dysmotility and represent prodromal events in AD.

## 1. Introduction

Ageing-related cognitive disorders, including age-associated memory impairment (AAMI), mild cognitive impairment (MCI) and senile dementia, have become common health threats to the elderly population. MCI manifests as an intermediate condition between age-related cognitive decline and dementia and represents a prodromal stage before the development of Alzheimer’s disease (AD) [1,2]. AD is one of the most common neurodegenerative disorders, characterised by a progressive memory decline, cognitive dysfunctions, amyloid β1-42 (Aβ) plaque accumulation, neurofibrillary tangle formation and occurrence of neurogenic/inflammatory responses in the central nervous system (CNS) [3].

Of interest, over the last years, alterations of the enteric bacteria-neuro-immune network have been proposed to be involved in the onset of AD and related gut dysfunctions [4]. In particular, changes in gut microbiota composition and impairments of the intestinal epithelial barrier (IEB) could determine the accumulation of enteric Aβ and phosphorylated tau (p-tau) proteins, which, in turn, could trigger enteric and peripheral neurogenic/inflammatory responses, and contribute to bowel motor disturbances as well as CNS neuroinflammation and neurodegeneration via gut–brain ascending pathways [4,5]. Indeed, both pre-clinical and human studies have shown that AD is associated with several changes in gut microbiota composition, signs of enteric inflammation as well as colonic accumulation of Aβ and p-tau tangle-like structures, which could lead to intestinal motor dysfunctions [6,7,8,9]. In particular, transgenic AD mice were found to display gut dysbiosis, intestinal Aβ and amyloid protein precursor (APP) accumulation, activation of intestinal inflammatory pathways, neuronal loss and enteric glial activation in the early stages of AD [10,11]. Other authors reported rearrangements of enteric neuronal coding, characterised by a decrease in neuronal nitric oxide synthase (nNOS) and choline acetyltransferase (ChAT) in mice with early AD [12]. However, whether colonic dysmotility, enteric AD-related protein accumulation and colonic inflammation represent the earliest events in AD, occurring in the prodromal stage of the disease, presently remains unclear. Likewise, the relationship between onset of cognitive impairment and bowel inflammation, colonic dysmotility and AD-related proteins before the full development of brain pathology remains to be explored in depth.

The present study was designed to examine the relationship between the onset of cognitive deficiencies and colonic dysmotility/inflammation, enteric depositions of AD-related proteins (Aβ, tau, p-tau, α-synuclein (α-syn) and their heterocomplexes) in the prodromal phases of AD in the SAMP8 spontaneous AD model. The SAMP8 mouse is one of the accelerated senescence strains that develop spontaneously early learning and memory deficiencies, with similar features to those observed in AD [13,14]. It is an excellent model for studying age-dependent cognitive decline associated with MCI and the subsequent development of AD [15,16]. Indeed, SAMP8 mice develop early learning and memory deficiencies since their young age (3–5 months), progressing then toward the development of full AD pathology (8–12 months), where they display the main pathophysiological and clinical features of AD, including the deposition of Aβ 1–40 or 1–42 proteins in hippocampal granules; hyperphosphorylation of tau protein; increase in α-syn, presenilin, oxidative damage, glutamate and nNOS levels; and decrease in ChAT activity [14]. Thus, owing to these features, the SAMP8 mouse can be regarded as an extremely valuable model for investigating intestinal symptoms and alterations in the prodromal stages of AD [14,17,18,19,20]. On these bases, since our intent was to examine whether the onset of cognitive impairment was associated with the occurrence of intestinal symptoms, we decided to perform a timing at four, six and eight months of age in SAMP8 mice to examine the onset of intestinal dysfunctions in the earliest stages of AD (MCI) before the full development of brain pathology. Gaining knowledge in this setting is critical for a better understanding of the mechanisms underlying bowel dysfunctions in AD and designing rational therapeutic approaches.

## 2. Results

### 2.1. Evaluation of Cognitive Functions (MWM Test)

**Figure 1:**
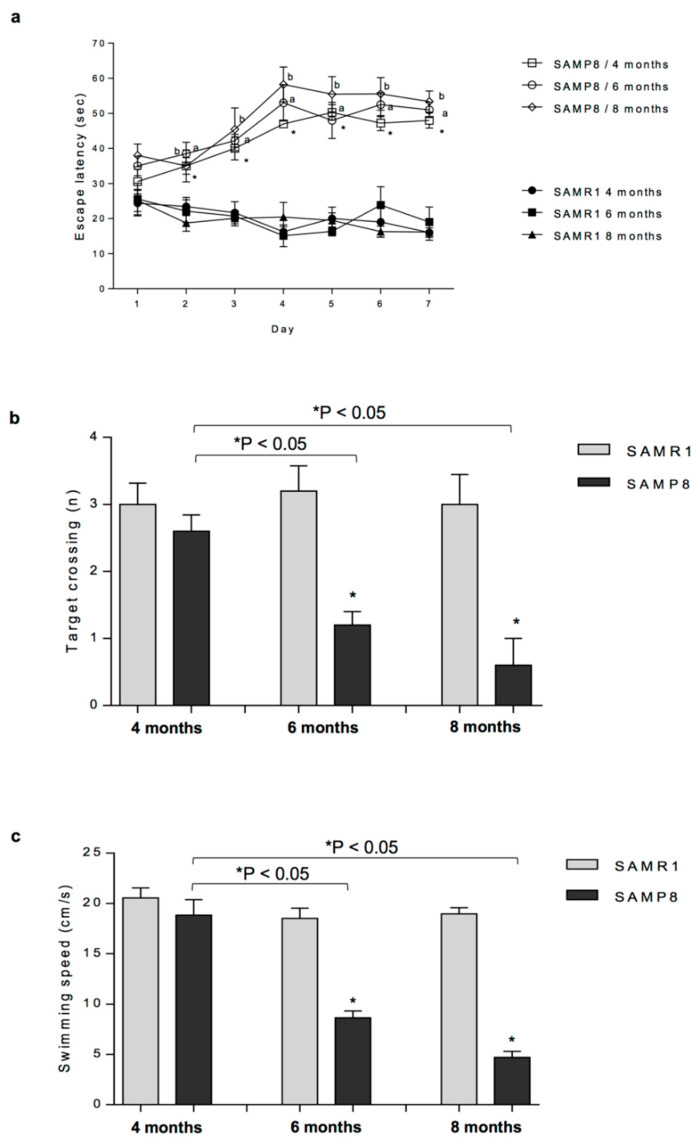
Cognitive performance of SAMR1 and SAMP8 mice at four, six and eight months of age, during the training and probe trial session of the Morris water maze test: (**a**) escape latency in SAMR1 and SAMP8 mice at four, six and eight months of age, during seven consecutive days of Morris water maze test training; (**b**) number of target crossings; and (**c**) swimming speed. Data are expressed as mean ± S.E.M. from eight animals. * *p* < 0.05, aP < 0.05, bP < 0.05, significant differences vs. age-matched SAMR1. Statistics: two-way ANOVA followed by post hoc analysis with Fisher LSD test (for paired data).

During the training test, at four, six and eight months SAMP8 mice displayed a significant increase in the escape latency time, starting from the second day of the test, as compared with SAMR1 mice (Figure 1a). In particular, even though SAMP8 animals showed an increasing trend in escape latency time, while SAMR1 mice displayed a decreasing trend, no significant differences were observed among the training days both in SAMP8 and SAMR1 animals (Figure 1a).

During the probe trial, the number of target crossings decreased significantly in SAMP8 mice at six and eight months, as compared with controls (SAMR1) and SAMP8 animals at four months (Figure 1b).

Swimming speed decreased significantly in SAMP8 mice at six and eight months, as compared with age-matched SAMR1 animals and SAMP8 at four months (Figure 1c).

Overall, cognitive impairments occurred in SAMP8 starting from six months of age, which reflects the prodromal AD phase, thus confirming that SAMP8 mice develop spontaneously alterations of spatial learning and memory, as compared with control SAMR1 mice.

### 2.2. Faecal Output and Feeding Behaviour

**Figure 2:**
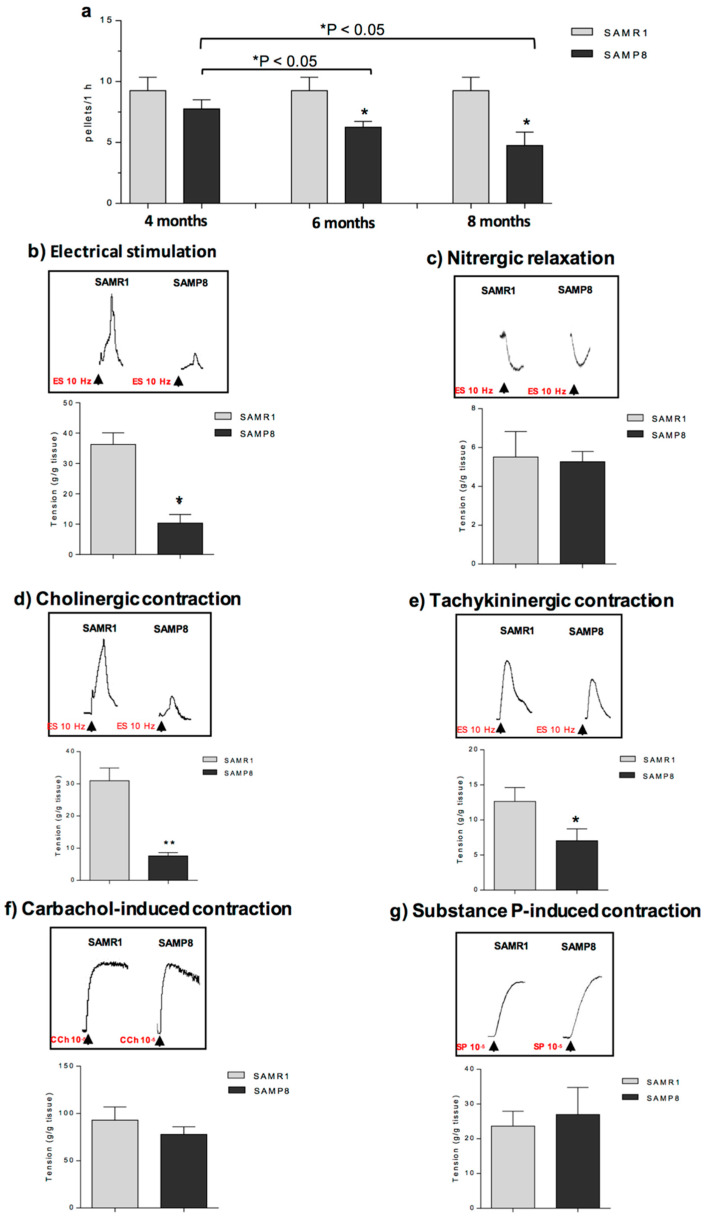
Faecal output and in vitro colonic contractile responses. (**a**) Faecal output expressed as number of pellets in 1 h in SAMR1 or SAMP8 mice at six months of age. Effects of electrical stimulation (ES, 10 Hz) on contractile activity of colonic longitudinal smooth muscle preparations isolated from SAMR1 or SAMP8 mice at six months of age: (**b**,**c**) colonic tissues maintained in standard Krebs solution; (**d**) colonic tissues maintained in Krebs solution containing L-NAME (100 μM), guanethidine (10 μM), L-732,138, (10 μM), GR159897 (1 μM) and SB218795 (1 μM) to record cholinergic contractions; (**e**) colonic tissues maintained in Krebs solution containing L-NAME (100 μM), guanethidine (10 μM), atropine sulphate (1 μM), GR159897 (1 μM) and SB218795 (1 μM) to record NK1-mediated tachykininergic contractions; (**f**) colonic preparations maintained in Krebs solution containing tetrodotoxin (TTX) (1 μM) and stimulated with CCh (10 μM) to record cholinergic contractions mediated by muscarinic receptors; and (**g**) colonic specimens maintained in standard Krebs solution, added with TTX and stimulated with exogenous SP (1 μM) to record contractions mediated by NK1 tachykininergic receptors. (**b**–**g**) Tracings in the inset on the top of panels display the contractile responses to ES or CCh or exogenous SP. Each column represents the mean ± S.E.M. from eight animals. * *p* < 0.05, ** *p*< 0.01, vs. SAMR1 significant difference versus SAMR1. Statistics: (**a**) two-way ANOVA followed by post hoc analysis with the Fisher LSD test (for paired data); or (**b**–**g**) Student t test (for unpaired data).

SAMP8 animals displayed a significant decrease in stool frequency starting from six months of age, as compared with age-matched control animals, while no differences were observed in SAMP8 and SAMR1 animals at four months of age (Figure 2a). These data indicate that AD is associated with a decrease in in vivo colonic transit.

During the observation period, the frequency and amount of food intake was about 4 g/day/mouse, with values not differing significantly between SAMP8 and SAMR1 mice.

### 2.3. In Vitro Colonic Contractile Activity

To verify whether the decreased in vivo colonic motility in SAMP8 mice can result from changes in enteric neurotransmissions, the patterns of in vitro colonic motor pathways were assessed.

In colonic longitudinal muscle preparations maintained in standard Krebs solution, the contractions evoked by ES accounted for 36.2 ± 3.8 g/g tissue for SAMR1 mice. In preparations from SAMP8 mice, electrically evoked contractions were significantly reduced (10.34 ± 2.8 g/g tissue) (Figure 2b).

In colonic preparations maintained in Krebs solution added with guanethidine, atropine, L-732,138, GR159897 and SB218795, the electrically evoked L-NAME-sensitive nitrergic relaxations did not differ between SAMP8 and SAMR1 animals (5.5 ± 1.3 and 5.3 ± 0.5 g/g tissue, respectively) (Figure 2c), indicating no changes in inhibitory nitrergic neurotransmission.

In colonic preparations maintained in Krebs solution added with L-NAME, guanethidine, L-732,138, GR159897 and SB218795, the electrically evoked atropine-sensitive cholinergic contractions were significantly reduced in the SAMP8 group as compared with SAMR1 (7.6 ± 1.1 and 30.9 ± 3.9 g/g tissue, respectively) (Figure 2d), thus suggesting an impairment of enteric cholinergic neuromuscular pathway.

In colonic preparations maintained in Krebs solution containing L-NAME, guanethidine, atropine, GR159897 and SB218795, the ES-induced NK_1_-mediated contractions were significantly reduced in SAMP8 mice, as compared with SAMR1 (12.6 ± 1.9 and 7.1 ± 1.7 g/g tissue, respectively) (Figure 2e), indicating a decrease in enteric tachykininergic NK_1_-mediated contractions.

The stimulation by CCh or exogenous SP of colonic preparations from SAMP8 or SAMR1 mice elicited contractions of similar magnitude (77.9 ± 7.9 and 93.0 ± 13.9 g/g tissue, respectively, for CCh-induced stimulation; 27.0 ± 7.7 and 23.7 ± 4.2 g/g tissue, respectively, for SP-induced contraction) (Figure 2f,g), suggesting no alterations in both CCh- and SP-induced myogenic contractions.

Overall, these findings showed that SAMP8 mice displayed a decrease in neurogenic cholinergic and tachykininergic colonic contractions while electrically evoked inhibitory nitrergic responses were unchanged, resulting in an impaired overall colonic propulsive motility.

### 2.4. AD-Related Proteins and Their Heteroaggregates

Next, the accumulation of AD-related proteins was verified in colonic tissues as a further sign of bowel involvement in AD pathology.

**Figure 3:**
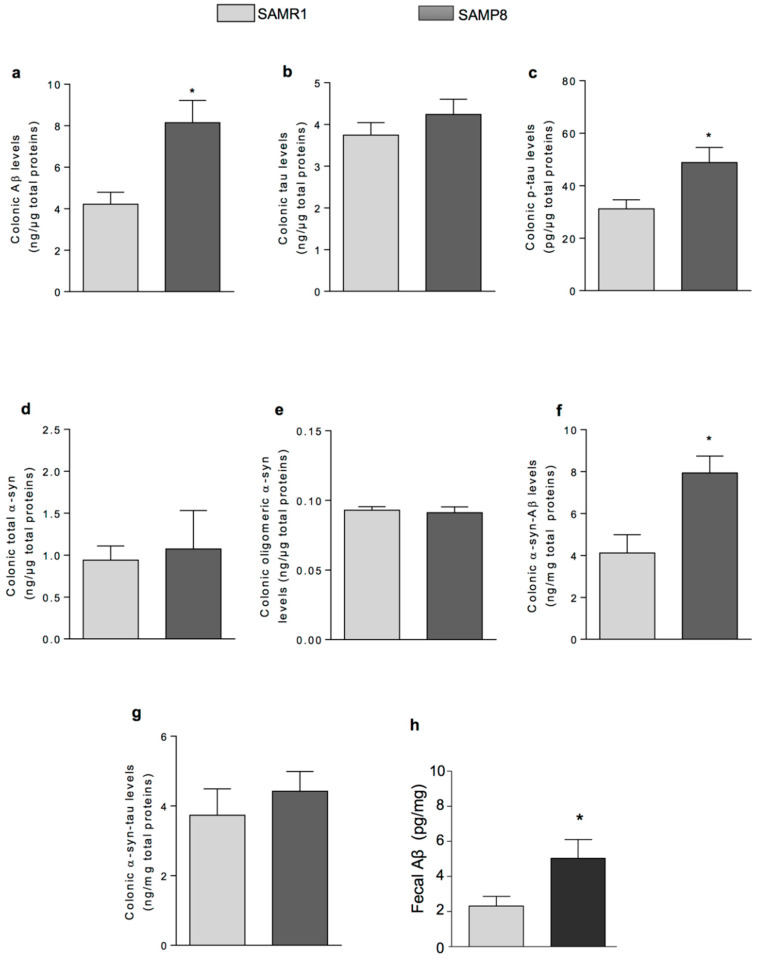
Quantitative detection of neurodegeneration-related proteins in colonic tissues and faecal specimens. The levels of: colonic Aβ (**a**); tau (**b**); p-tau (**c**); total α-syn (**d**); oligomeric α-syn (**e**); α-syn-Aβ (**f**); and α-syn-tau (**g**) were determined in SAMP8 and SAMR1 mice at six months by specific immunoenzymatic assays. (**h**) The levels of faecal Aβ were quantified in SAMR1 and SAMP8. Data are expressed as mean ± S.E.M. from eight animals. * *p* < 0.05, significant difference versus SAMR1. Statistics: Student *t*-test (for unpaired data).

Colonic Aβ concentrations were significantly higher in SAMP8 with respect to SAMR1 animals (Figure 3a). Colonic total tau did not differ in SAMR1 and SAMP8 (Figure 3b). Nevertheless, the pathogenic form of tau, i.e., its phosphorylated form, was significantly higher in SAMP8 than SAMR1 mice (Figure 3c).

The colonic levels of both total and oligomeric α-syn were similar in control and SAMP8 mice (total α-syn; oligomeric α-syn) (Figure 3d,e, respectively).

The amount of α-syn-Aβ heterocomplexes in the colon from SAMP8 mice was significantly higher than in age-matched SAMR1 (Figure 3f). By contrast, comparable levels of α-syn-tau were detected in SAMP8 and age-matched SAMR1 mice (Figure 3g).

The amount of Aβ was measured in faecal samples too. Faecal Aβ levels in SAMP8 mice were higher as compared with age-matched SAMR1 (5.12 ± 1.03 and 2.34 ± 0.72 pg/mg, respectively) (Figure 3h). These data demonstrate that Aβ accumulates in faecal samples from SAMP8 mice.

### 2.5. Mitochondrial Activity in Colonic Tissues

**Figure 4:**
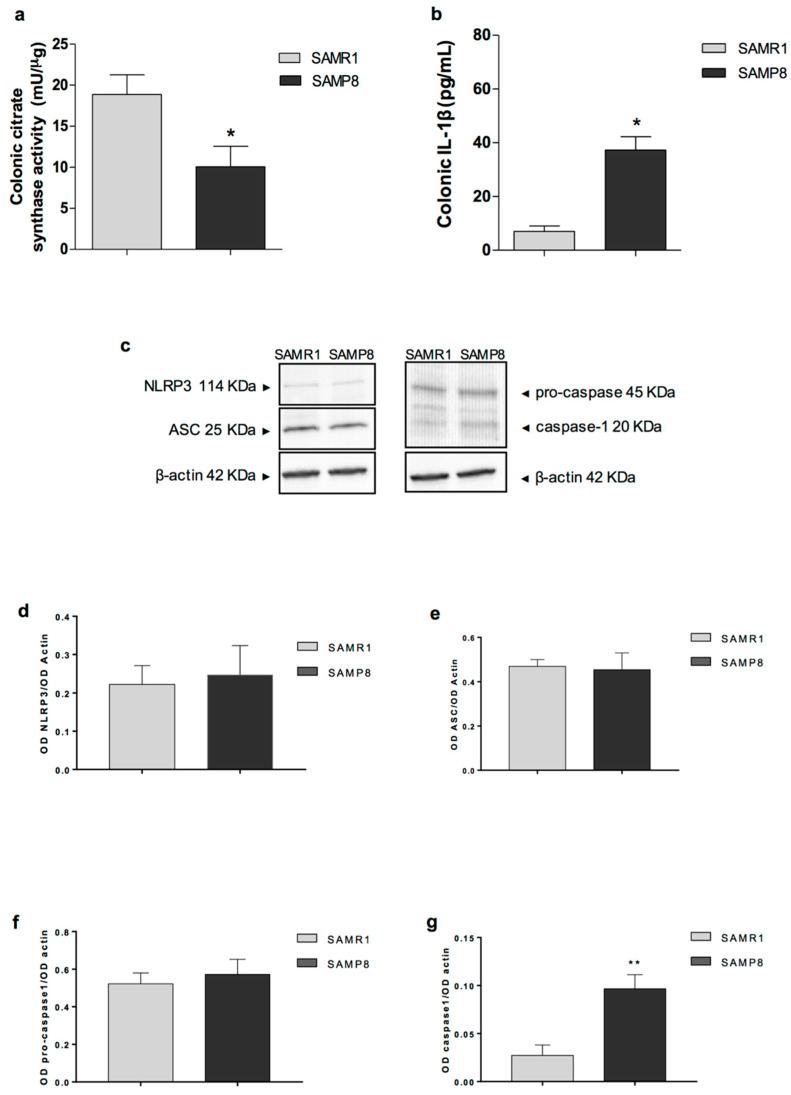
Mitochondrial activity and inflammation in colonic specimens: (**a**) citrate synthase in colonic tissues from SAMR1 and SAMP8 animals at six months of age; (**b**) IL-1β levels in colonic tissues from SAMR1 and SAMP8 animals at six months of age; (**c**) representative immunoblot from four mice per group; and (**d**–**g**) densitometry of Western blot results for NLRP3, ASC, pro-caspase-1 and cleaved caspase-1 in SAMR1 and SAMP8 mice. Expression was normalised against β-actin. Data are expressed as mean ± S.E.M. from eight animals. * *p* < 0.05, ** *p* < 0.01, significant differences versus age-matched SAMR1. Statistics: Student’s *t*-test (for unpaired data).

Citrate synthase activity in SAMP8 mice was significantly decreased as compared with SAMR1 mice (18.9 ± 2.4 and 10.1 ± 2.5 mU/µg, respectively) (Figure 4a), suggesting that the senescence processes deeply reduce the metabolic activity of the colon.

### 2.6. IL-1β Levels in Colonic Tissues

In colonic tissues from SAMR1 animals, the levels of the pro-inflammatory cytokine IL-1β accounted for 5.2 ± 1.1 pg/mg tissue (Figure 4b). In colonic specimens from SAMP8 mice, IL-1β levels were significantly higher (15.9 ± 1.6 pg/mg tissue) as compared with age-matched SAMR1 mice (Figure 4b).

### 2.7. Expression of NLRP3, ASC, Pro-Caspase-1 and Cleaved Caspase-1 in Colonic Tissues

To further investigate the activation of the pro-inflammatory signalling (i.e., IL-1β,) we evaluated the expression of inflammasome components, including NLRP3, ASC and caspase-1, in colonic tissues from SAMP8 mice [21]. The colonic expression of NLRP3 (114 KDa), ASC (25 KDa) and pro-caspase-1 (45 KDa) did not differ between SAMR1 and SAMP8 mice (Figure 4d–f). The expression of cleaved caspase-1 (p20, an autoprocessed fragment of caspase-1) was significantly higher in colonic tissues from SAMP8 as compared with SAMR1 mice (0.097 ± 0.015 and 0.027 ± 0.011 OD caspase-1/OD actin, respectively) (Figure 4g). Globally, the increase in caspase-1 cleavage, along with the increase in IL-1β levels, indicates the activation of the well-established pattern of canonical inflammasome pathway [22].

### 2.8. Distribution and Density of Eosinophils in Colonic Tissues

**Figure 5:**
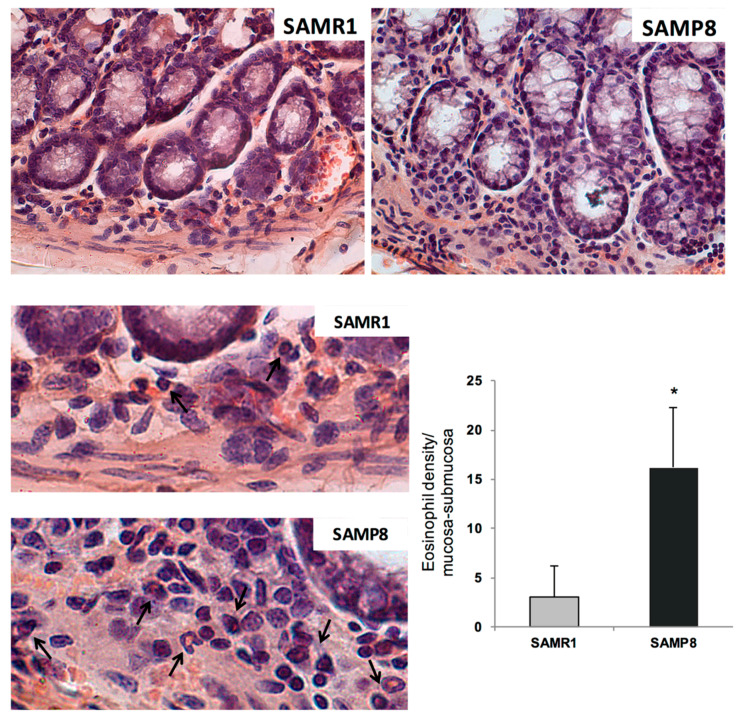
Representative pictures of Congo Red-stained eosinophils (black arrows) in colonic sections from SAMR1 and SAMP8 at six months of age. Original magnification: 40×. The column graph displays the mean values of eosinophil density per square millimetre of tunica mucosa/submucosa areas (cells/mm^2^) ± S.E.M. from eight animals. * *p* < 0.05. Statistics: Student’s *t*-test (for unpaired data).

The increase in eosinophil distribution and density in the gut wall is regarded as an index to estimate the presence of an enteric inflammatory condition [23]. Eosinophils, which were frequently found within the tunica mucosa and submucosa of the normal colon, were significantly more represented in SAMP8 mice, as compared with SAMR1, which displayed also sporadic eosinophils in the tunica muscularis (Figure 5).

### 2.9. Morphological Changes in Intestinal Mucosal Barrier: Claudin-1 Expression and Distribution

**Figure 6:**
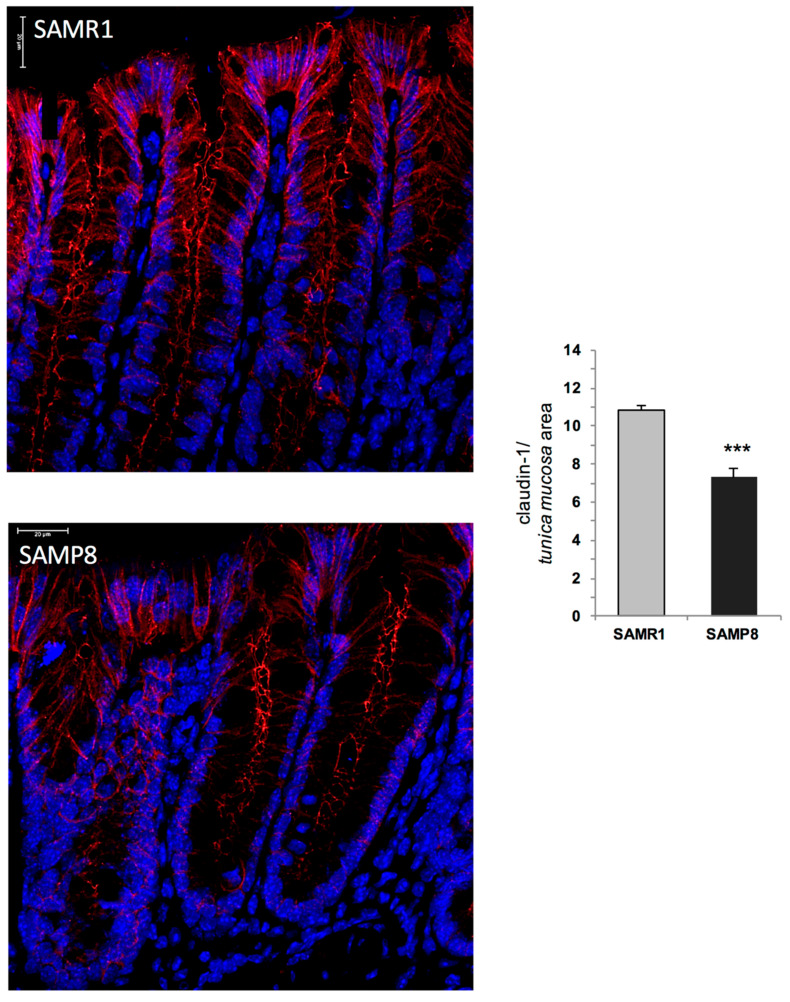
Representative photomicrographs of claudin-1 immunostaining in the cross-sectioned colon from SAMR1 and SAMP8 mice at six months of age. Original magnification 63×. The column graph displays the mean values of the percentage of positive pixels (PPP) within the respective tissue area examined ± S.E.M from eight animals. Scale bar 20 µm *** *p* < 0.001. Statistics: Student’s *t*-test (for unpaired data).

In SAMR1 mice, lining colonic epithelial cells expressed an abundant amount of claudin-1, one of the main components of tight junctions (Figure 6). In SAMP8 animals, the colonic epithelium displayed a significant decrease in claudin-1 immunostaining (Figure 6).

### 2.10. In Vitro Assays on NLRP3 Inflammasome and Mitochondrial Function

**Figure 7:**
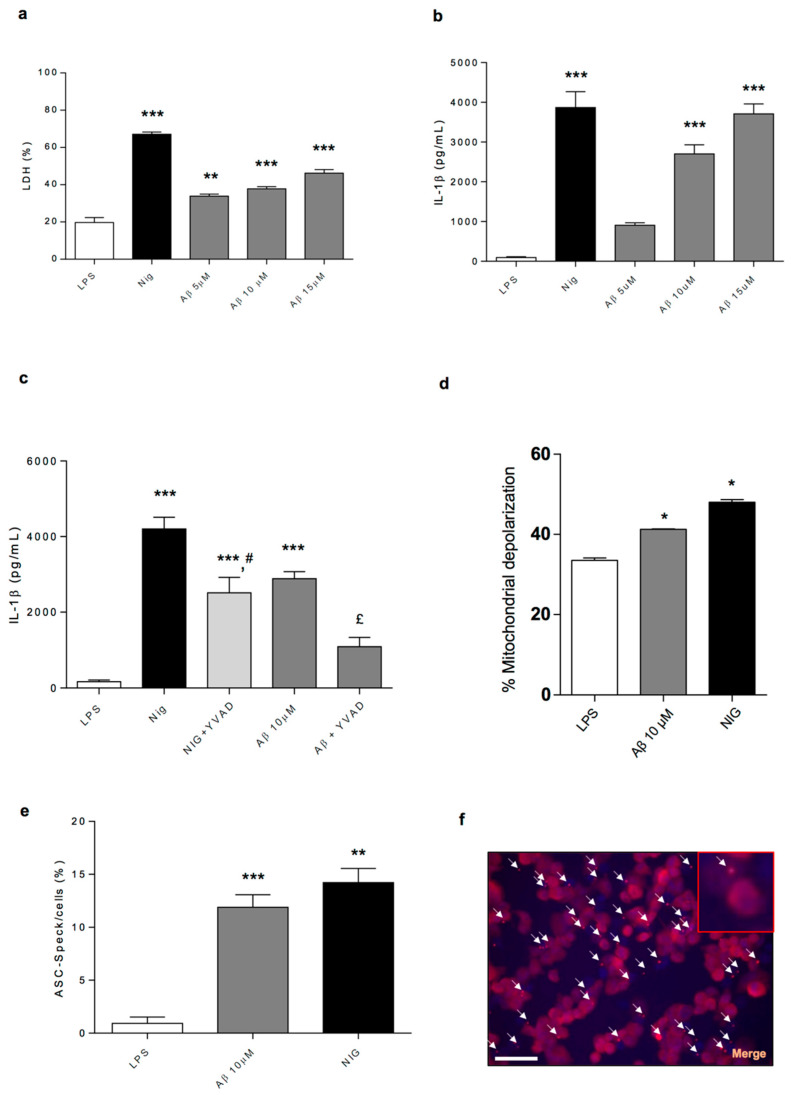
(**a**) LDH in supernatants from wild type THP-1 treated with LPS (1 g/mL, 4 h) and LPS plus Nig (10 μM, 1 h) or Aβ (5, 10 and 15 μM, 6 h). (**b**) IL-1β levels in supernatants from LPS-primed THP-1 cells treated with Nig or Aβ. (**c**) IL-1β levels in supernatants from LPS-primed THP-1 cells treated with Nig or Aβ, 10 μM, 6 h in the presence or the absence of caspase-1 inhibitor (YVAD, 100 µM). (**d**) Mitochondrial depolarisation in supernatants from LPS-primed THP-1 cells treated with or Aβ. (**e**) Immunofluorescence analysis of ASC in LPS-primed (1 g/mL, 4 h) THP-1 cells treated with Nig or Aβ. The number of ASC specks are quantified and expressed as the percentage of specks per cell number. (**f**) Representative immunofluorescence images of ASC expression in LPS-primed THP-1 treated with Nig or Aβ. Scale bar = 50 μM. Each column represents the mean ± S.E.M. value obtained from 4 separate experiments. ** *p* < 0.01, significant difference versus LPS-primed THP-1 cells; *** *p* < 0.001, significant difference versus LPS-primed THP-1 cells; # *p* < 0.05, significant difference versus nigericin; ^£^ *p* < 0.01, significant difference versus Aβ, * *p* < 0.05, significant difference versus LPS. Statistics: One-way ANOVA followed by post hoc analysis with Student-Bonferroni test (for paired data).

*Cell death*: In LPS-primed THP-1 cells, LDH release accounted for 20% of total lysed cells (Figure 7a). Incubation with nigericin (+47.4%) or Aβ (+14.9%, +19.2% and +28.1%, respectively) induced cell death in LPS-primed THP-1 cells (Figure 7a).

*IL-1β processing and release*: In LPS-primed THP-1 cells, IL-1β levels accounted for 104 ± 25.6 pg/mL (Figure 7b). Incubation with nigericin or Aβ stimulated IL-1β release (Figure 7b,c). Treatment with caspase-1 inhibitor (YVAD) reduced significantly the processing and release of IL-1β induced by nigericin or Aβ (Figure 7c). In LPS-primed ASC^-/-^ THP-1 cells, IL-1β release accounted for 12.9 ± 1.3 pg/mL. The treatment with nigericin or Aβ did not stimulate IL-1β processing and release (15.6 ± 1.6 and 16.9 ± 1.7 pg/mL, respectively).

*ASC Speck Detection and Quantification*: ASC is an adaptor protein required for the activation of NLRP3 inflammasomes [24], and, upon inflammasome assembly, its presence within the complex is readily visualised inside cells by its oligomerisation and appearance of large aggregates, designated as specks [24]. Incubation of LPS-primed THP-1 cells with nigericin increased the numbers of ASC specks (+13.5%) (Figure 7e,f and Figure S1). Likewise, treatment with Aβ was able to promote ASC oligomerisation (+10.3%) (Figure 7e,f and Figure S1).

*Mitochondrial potential*: The incubation of THP-1 cells with LPS plus nigericin increased significantly the number of depolarised cells as compared to LPS-treated cells (+14.6%) (Figure 7d). Likewise, the incubation with 10 µM Aβ was associated with significant mitochondrial membrane depolarisation, as compared with LPS-treated cells (+7.7%) (Figure 7d).

## 3. Discussion

Several lines of evidence suggest that, in the early stages of AD, changes in gut microbiota composition, impaired IEB, AD-related protein accumulation in intestinal tissues and enteric inflammation could contribute to CNS pathology and related intestinal dysfunctions [4]. However, current evidence does not allow establishing clear relationships among colonic dysmotility; enteric AD-related protein accumulation, including Aβ, p-tau, α-syn and their heterocomplexes; bowel inflammation; and AD pathology since the earliest stages of the disease. In this context, our purpose was to examine the occurrence of bowel dysmotility, enteric AD-related protein accumulation, colonic inflammation and IEB impairment in a murine model of accelerated senescence (SAMP8 mouse) in the early phase of AD, preceding the full development of brain pathology. The SAMP8 mouse develops spontaneously early learning and memory deficits, with similar features to those observed in AD patients. Of interest, the SAMP8 mouse, which develops a severe disease after eight months of age, can be an extremely valuable model to investigate intestinal symptoms in the prodromal stage of AD [13,14,25].

As a first step, we attempted to determine a possible relationship between the onset of cognitive and motor impairments and colonic dysmotility in SAMP8 mice. To pursue this goal, we performed the MWM test and evaluated the faecal output in SAMP8 and SAMR1 animals at four, six and eight months of age. Our results show that SAMP8 mice displayed an impairment of cognitive and motor functions along with a significant decrease in faecal output starting from six months of age, thus indicating that, in SAMP8 animals, the alterations of colonic motility appear in the prodromal phase of AD, before the full development of CNS pathology. Consistently with our findings, several studies have shown that both patients with MCI and early AD are characterised by infrequent bowel movements and constipation [26,27].

Based on results from in vivo experiments, we decided to focus the attention on SAMP8 animals at six months of age, in order to examine the mechanisms underlying the intestinal motor dysfunctions associated with the onset of the cognitive and motor symptoms, before the full development of AD. Therefore, in the second part of the study, to verify whether the impaired in vivo colonic motility in SAMP8 mice at six months of age might result from changes in enteric neurotransmission, we examined the patterns of in vitro excitatory (cholinergic and tachykininergic) and inhibitory (nitrergic) motor pathways. Our results show that electrically evoked cholinergic and tachykininergic contractions of colonic muscle preparations from SAMP8 mice were significantly decreased, while electrically evoked inhibitory nitrergic responses were unchanged, resulting in an impaired overall colonic propulsive motility. These findings support the view that the cognitive decline is associated with altered excitatory control of colonic motility, thus providing new insights into the pathophysiological mechanisms underlying the occurrence of bowel dysfunctions in the prodromal stages of AD. Subsequently, to verify whether the decrease in cholinergic and tachykininergic colonic contractions could depend on changes in the density of muscarinic or NK_1_ tachykininergic receptors on smooth muscle cells, we examined the myogenic colonic contractions through direct stimulation of muscarinic and NK_1_ receptors with carbachol and exogenous substance P, respectively. Our results show no changes in colonic myogenic cholinergic and NK_1_-mediated tachykininergic contractions, thus suggesting that the impairments of colonic excitatory contractile responses could be ascribed to an impairment of both cholinergic and tachykininergic excitatory neurotransmission.

Taken together, our results provide the first demonstration that rearrangements of enteric excitatory neuronal motor pathways could contribute to colonic dysmotility occurring in SAMP8 mice in the prodromal stages of AD. Of note, these results are in line with a recent study showing that APP/presenilin 1 (PS1) transgenic AD mice displayed a remodelling of enteric neuronal coding, characterised by a decrease in nNOS and ChAT [12]. However, these authors evaluated the density of nitrergic and cholinergic neurons, omitting the assessment of colonic in vitro motor activity.

Of note, several lines of evidence suggest that enteric AD-related protein accumulation, including Aβ and p-tau proteins, shape the immune/inflammatory responses that, in turn, could contribute to bowel motor dysfunctions since the earliest stages of AD [5]. Indeed, enteric Aβ and p-tau protein aggregates and increased faecal calprotectin levels have been observed in AD patients at different stages of the disease [6,7].

Based on the above knowledge, in the third part of the present study, we focused our attention on investigating the presence of AD-related proteins (i.e., α-syn, tau and Aβ) and their heteroaggregates, including α-syn-tau and α-syn-Aβ, as well as inflammation in colonic tissues from SAMP8 mice. In addition, we evaluated the faecal levels of Aβ which is the dominant variant of Aβ deposits in the human brain [28]. SAMP8 mice displayed an increase in faecal Aβ as well as colonic Aβ and p-tau levels, as compared with controls. These findings reflect Aβ and p-tau accumulation detected in brain tissues from SAMP8 at six months in previous studies, and suggest that AD-related protein deposits in intestinal tissues correlate with those detected in the brain from SAMP8 mice at early stages of the disease [25,29]. By contrast, no variation of colonic total and oligomeric α-syn levels were found when comparing SAMP8 and SAMR1 animals. These data suggest that α-syn is not involved in the enteric AD-protein accumulation in the SAMP8 model. With regard to the assessment of AD-related protein heterocomplexes, we found an increase in the levels of α-syn-Aβ and α-syn-tau deposits in colonic tissues from SAMP8, suggesting that enteric Aβ and tau accumulation could increase their interaction with α-syn. These results are in line with our previous findings showing increased levels of α-syn-Aβ and α-syn-tau heterocomplexes in the brain and red blood cells from SAMP8 mice at six months [25]. Moreover, we observed an increase in IL-1β levels in colonic tissues from SAMP8 animals, suggesting the occurrence of inflammatory responses in the large bowel of early AD mice. Overall, these observations suggest that, in early AD, the accumulation of AD-related proteins and their heterocomplexes could promote neurogenic/inflammatory responses, which, in turn, could contribute to bowel motor disturbances.

Of note, the accumulation of AD-related proteins and their heteroaggregates could promote mitochondrial dysfunctions (a hallmark of Aβ-induced neuronal toxicity in AD) in the colon of SAMP8 mice [30]. Therefore, we went on to evaluate the citrate synthase activity, referred to as a suitable marker of mitochondrial activity, which decreases dramatically in several organs and tissues during ageing [31], in colonic tissues from SAMP8 animals. This rate-limiting mitochondrial enzyme is involved in the first step of the Krebs cycle and catalyses the condensation reaction of the acetate residue from acetyl coenzyme A and oxalacetate to form citrate in mitochondria [32]. In our study, the citrate synthase activity was significantly decreased in colonic tissues from SAMP8 animals at six months. Such a decrease could involve different intestinal cell types, including intestinal epithelial cells (i.e., Lgr5+ crypt based columnar cells characterised by high basal mitochondrial activity) [33,34] and intestinal innate immune/inflammatory cells, including macrophages, regarded as immune sentinels which sense several pathological stimuli, including Aβ, that, in turn, is known to activate inflammatory pathways and mitochondrial dysfunction [35,36].

These findings suggest that enteric AD protein accumulation, colonic inflammation, and mitochondrial dysfunction are among the earliest events in AD that could contribute to bowel dysmotility. The presence of bowel inflammation in SAMP8 mice was further corroborated by an increase in eosinophil within the colonic tunica mucosa and submucosa. These results are in line with previous studies, showing an increase in Aβ and p-tau protein expression, as well as immune/inflammatory cell activation in intestinal tissues from TgCRND8 and APP/PS1 mice (genetic models of AD) since the early stages of AD [11,37].

It is noteworthy that the accumulation of Aβ proteins, mitochondrial dysfunction and the increase in IL-1β levels in colonic tissues from SAMP8 mice suggest the involvement of the NLRP3 inflammasome multiprotein complex in the onset of enteric inflammation [21]. In this respect, we evaluated the expression of inflammasome components, including NLRP3, ASC, and caspase-1 in colonic tissues from SAMP8 mice, and we found a significant increase in caspase-1 cleavage, while no changes in NLRP3 and ASC expression were detected. The increase in caspase-1 cleavage, along with the increase in Aβ proteins, mitochondrial dysfunction, and IL-1β levels, indicate the activation of the well-established pattern of canonical inflammasome pathway [22]. Interestingly, a similar picture has been observed in brain tissues from AD mice, where NLRP3 activation and Aβ protein deposition, mitochondrial dysfunction, and IL-1β were detected [38].

Based on the present findings, it is conceivable that, in early AD, the accumulation of Aβ proteins promotes mitochondrial dysfunction and inflammasome activation, which, in turn, shaping neurogenic/inflammatory responses, could contribute to bowel dysmotility. In support of this view, the accumulation of Aβ proteins in AD has been proposed to contribute to mitochondrial dysfunction as well as to activate the NLRP3 inflammasome complex in the CNS [39]. In an attempt of confirming this hypothesis, in the fourth part of the present study, we went on to characterise in vitro the molecular mechanisms through which Aβ, regarded as the dominant variant of AD-related proteins, can determine NLRP3 inflammasome activation and mitochondrial dysfunction in immune cells. To pursue this goal, we performed experiments in the LPS-primed PMA-differentiated THP-1 cell line, an established model to investigate monocyte/macrophage functions. In particular, we tested the ability of Aβ of inducing the release of IL-1β through direct activation of NLRP3 inflammasome and to alter mitochondrial functions. Interestingly, our results show that Aβ was able to stimulate NLRP3 activation and induce IL-1β release in a concentration-dependent fashion in THP-1 cells through the stimulation of ASC oligomerisation, a pivotal step in NLRP3 activation. In support of these findings, Aβ did not induce the release of IL-1β in ASC^-/-^ THP-1 cells or in WT cells pre-treated with a caspase-1 inhibitor. These findings corroborate previous findings indicating that Aβ accumulation promotes the release of IL-1β through the direct activation of the NLRP3 inflammasome complex in immune cells [39]. In addition, the Aβ treatment of THP-1 cells decreased the mitochondrial potential, thus suggesting that Aβ is involved also in the mechanisms leading to alteration of mitochondrial activity.

Based on the above findings, it is conceivable that, in early AD mice, the enteric accumulation of AD-related proteins, with particular regard for Aβ, determines NLRP3 activation and mitochondrial dysfunctions, which, in turn, promote the occurrence of enteric neurogenic/inflammatory conditions that could contribute to alterations of bowel motility. However, whether the Aβ-induced NLRP3 activation, besides shaping the immune/inflammatory responses, contributes also to alter the enteric neuronal pathways, or whether both events occur concomitantly, remains to be clarified. In addition, studies aimed at evaluating the specific enteric neuronal and immune/inflammatory cell types (i.e., myenteric neurons, enteric glial cells and macrophages) involved in the onset of enteric inflammation associated with AD would be required. The occurrence of enteric inflammation, besides contributing to bowel motor dysfunctions, could alter the IEB, with consequent alterations of enteric permeability [40]. Therefore, in the last part of the study, we evaluated the morphological changes of the intestinal mucosal barrier in SAMP8 mice. In particular, we focused our attention on claudin-1 protein, a pivotal tight junction component involved in the maintenance of IEB integrity [41]. Our results showed a decreased expression of claudin-1 in SAMP8 mice, suggesting an impairment of IEB in early AD animals. Such a decrease could contribute to bacterial translocation into the intestinal mucosa, with consequent chronicisation of the ongoing inflammatory response and further worsening of bowel motor dysfunctions [42]. A similar trend has been observed in patients with early Parkinson’s disease, characterised by alterations of occludin and zonulin-1 expression in colonic tissues [43,44]. Based on these findings, it is conceivable that the enteric inflammation might impair the IEB integrity, with consequent alterations of intestinal permeability, and further worsening of bowel motor dysfunctions. However, the correlation among enteric inflammation, impaired IEB, and bowel dysmotility requires confirmation by means of additional experimental approaches. In addition, since the NLRP3 inflammasome is expressed also in intestinal epithelial cells [22], further experiments aimed at evaluating whether Aβ protein can promote inflammasome activation in intestinal epithelial cells should be implemented.

## 4. Materials and Methods

### 4.1. Animals

SAMP8 (Senescence-Accelerated Mouse-Prone 8) mice (2 months old, 20–25 g body weight), a spontaneous genetic model of AD, and their control strain SAMR1 (Senescence-Accelerated Mouse-Resistant 1) (2 months old, 20–25 g body weight) were purchased from ENVIGO Srl (San Pietro al Natisone UD, Italy) and employed throughout the study.

The animals were fed with standard laboratory chow and tap water ad libitum, and were not employed for at least 1 week after their delivery to the laboratory. They were housed, one in a cage, in temperature-controlled rooms on a 12-h light cycle at 22–24 °C and 50–60% humidity. Standard diet (Altromin International, Germany; SD, TD.2018) provided 3.1 kcal/g, of which 18% as fats, 24% as proteins and 58% as carbohydrates. The feeding behaviour (frequency and amount) was assessed until the day before the sacrifice. At the end of study, animals were euthanised. Animals were housed, three in a cage, in temperature-controlled rooms on a 12-h light cycle at 22–24 °C and 50–60% humidity. Their care and handling were in accordance with the provisions of the European Community Council Directive 210/63/UE, recognised and adopted by the Italian Government. The experiments were approved by the Ethical Committee for Animal Experimentation of the University of Pisa and by the Italian Ministry of Health on february 25th 2016 (Authorisation No. 198/2016-PR).

The SAMP8 mouse is one of the accelerated senescence strains that develops spontaneously early learning and memory deficits, with similar features to those observed in AD [13,14]. Of note, the SAMP8 mouse displays the main clinical and pathophysiological features to those observed in AD patients, including Aβ 1–40 or 1–42 proteins in hippocampal granules, hyperphosphorylation of tau protein, increase in α-syn, presenilin, oxidative damage, glutamate levels, and nNOS, along with the decrease in ChAT activity [14].

SAMP8 and SAMR1 animals at four, six and eight months of age were subjected to Morris Water Maze (MWM) test in order to evaluate alterations of cognitive functions from the initial phases of early learning and memory deficiencies until the full development of AD. The day after the evaluation of cognitive and motor performances, animals were employed for the assessment of faecal output, as described below. One hour after the evaluation of faecal output, SAMP8 and SAMR1 animals at six months of age were euthanised and colonic specimens were dissected and processed for functional experiments and other assays, as described below.

### 4.2. Evaluation of Cognitive Functions

MWM test: The MWM uses a round pool (90 cm in diameter and 60 cm in height) filled with opaque water (26 ± 1 °C temperature). The pool was divided into four quadrants of equal area, designated arbitrarily as northeast (NE), southeast (SE), southwest (SW) and northwest (NW). A circular platform (10 cm diameter and 30 cm height) was placed in the centre of one quadrant. All external clues were constant for the spatial orientation of mice. A camera mounted directly above the centre of the round pool monitored animal movements. The camera image was digitalised and fed to a computerised tracking system that monitored and stored animal movements [45].

The MWM test consists of visible-platform acquisition training, hidden-platform training, and probe trial as previously reported [25]. The platform was in the same location for both visible-platform training and hidden platform training. In the acquisition training, the escape latency was assessed for each animal (time required to reach the platform). Mice were placed on the platform for 15 s before being released into the water. Mice were allowed to swim and find the visible platform within 60 s. Each animal was subjected to sessions of four trials every day for 2 days. After the daily trial, mice were returned to their home cages for resting. In the hidden-platform training, performed by submerging the platform 1.5 cm below the surface of the water, escape latency was evaluated over the next 5 days. Each animal was subjected to sessions of four trials every day. Finally, on the eighth day, the platform was removed from the tank for the probe trial. The number of target crossings the number of entries into the target quadrant, the time spent in the target quadrant where the platform was placed, the swimming speed and swim distance were assessed in 60 s. Data were expressed as raw values.

### 4.3. Evaluation of Faecal Output

Faecal output was recorded from 9:00 AM to 10:00 AM on each day. Each animal was removed from its home cage and placed in a clean plastic cage without food or water for 1 h. Stools were collected immediately after expulsion and placed in sealed tubes. At the end of the trial, the stools were counted and weighed (total weight), then dried overnight at 65 °C and weighed again to estimate the dry weight.

### 4.4. Recording of Colonic Contractile Activity

The contractile activity of colonic muscle preparations was recorded as previously described by [46]. Following euthanasia, the abdomen was immediately opened, the colon was removed and placed in Krebs solution. Segments of the colon were opened along the mesenteric insertion and cut along the longitudinal axis into strips of approximately 3 mm in width and 8 mm in length. The preparations were set up in organ baths containing Krebs solution at 37 °C, bubbled with 95% O_2_ + 5% CO_2_ and connected to isometric transducers (constant load = 0.5 g). Krebs solution had the following composition (mM): NaCl 113, KCl 4.7, CaCl_2_ 2.5, KH_2_PO_4_ 1.2, MgSO_4_ 1.2, NaHCO_3_ 25 and glucose 11.5 (pH 7.4 ± 0.1). The mechanical activity was recorded by BIOPAC MP150 (Biomedica Mangoni, Pisa, Italy). Each preparation was allowed to equilibrate for at least 30 min, with intervening washings at 10 min intervals. A pair of coaxial platinum electrodes was positioned at a distance of 10 mm from the longitudinal axis of each preparation to deliver electrical stimulation by a BM-ST6 stimulator (Biomedica Mangoni, Pisa, Italy). Electrical stimuli (ES) were applied: 10-s single trains consisting of square wave pulses (0.5 ms, 30 mA). At the end of the equilibration period, each preparation was repeatedly challenged with ES, and the experiments started when reproducible responses were obtained (usually after two or three stimulations). The tension developed by each preparation was normalised by the wet tissue weight and expressed as grams per gram of wet tissue (g/g tissue).

Preliminary experiments were performed to select the appropriate ES frequency, as well as carbachol (CCh) or exogenous substance P (SP) concentration, which elicited submaximal contractions. These preliminary experiments allowed to select the frequency of 10 Hz and the concentration of 10 μM of CCh and 1 μM of SP.

### 4.5. Design of Experiments on Colonic Contractions

In the first series of experiments, electrically induced contractions were recorded from colonic preparations maintained in standard Krebs solution.

In the second set of experiments, electrically induced contractions were recorded from colonic preparations maintained in Krebs solution containing guanethidine (adrenergic blocker 10 μM), N-acetyl-l-tryptophan 3,5-bis(trifluoromethyl) benzylester (L-732,138, neurokinin NK_1_ receptor antagonist, 10 μM), 5-fluoro-3-[2-[4-methoxy-4-[[(R)-phenylsulphinyl]methyl]-1-piperidinyl]ethyl]-1H-indole (GR159897, NK_2_ receptor antagonist, 1 μM), (R)-[[(2-phenyl-4-quinolinyl)carbonyl]amino]-methyl ester benzeneacetic acid (SB218795, NK_3_ receptor antagonist, 1 μM) and atropine (muscarinic receptor antagonist, 1 μM) in order to evaluate the patterns of colonic contractions driven by nitrergic pathways.

In the third series, colonic specimens were maintained in Krebs solution containing Nω-nitro- L-arginine methylester (L-NAME, nitric oxide synthase inhibitor, 100 μM), guanethidine, L-732,138, GR159897 and SB218795 and contractions were elicited by ES in order to examine the patterns driven by excitatory cholinergic nerves.

The fourth series of experiments was designed to study the neurogenic NK_1_-mediated contribution to muscle contraction. For this purpose, colonic tissues were maintained in Krebs solution containing L-NAME, guanethidine, atropine sulphate, GR159897 and SB218795 and electrically evoked motor responses were recorded.

In the fifth set of experiments, colonic cholinergic contractions were evoked by direct pharmacological activation of muscarinic receptors located on smooth muscle cells. For this purpose, colonic preparations were maintained in Krebs solution containing tetrodotoxin (TTX, 1 μM) and stimulated with CCh (10 μM).

In the last series, tachykininergic NK_1_-mediated contractions were evoked by direct pharmacological activation of NK_1_ receptors located on smooth muscle cells. For this purpose, colonic specimens were maintained in standard Krebs solution, added with TTX and stimulated with exogenous SP (1 μM).

### 4.6. Collection of the Colon and Total Protein Quantification

Phosphate buffered saline (PBS) was added to colonic tissue specimens, and the samples were sonicated. A Bradford assay was performed to quantify the total proteins present in the samples. Then, sodium dodecyl sulphate (SDS) was added to samples to achieve a concentration of 100 μg/100 μL of total proteins.

### 4.7. Preparation of Oligomeric α-Syn and α-Syn Biotinylated Antibody

Recombinant α-syn was incubated in parafilm sealed tubes at 37 °C for 4 days in an Eppendorf Thermomixer under continuous mixing (1000 rpm) [47]. A reaction among Sulpho-NHS-LC-Biotin (Pierce, Rockford, IL, USA) (200 mg) and the 211 mouse monoclonal antibody (mAb) (Santa Cruz Biotechnology, USA) was allowed to prepare α-syn biotinylated antibody [48]. Then, the mixture was desalted on Bio-Spin-6 columns (BIO-RAD, Langford, UK) to eliminate excess uncoupled biotin [49,50,51].

### 4.8. Evaluation of Tissue and Faecal AD-related Protein Levels and Their Heterocomplexes

Colonic total α-syn: Colonic total α-syn was evaluated by a “home-made” sandwich enzyme-linked immunosorbent assay (ELISA) system [49,52]. First, 60 μL/well of full length rabbit polyclonal antibody to α-syn (SC-10717, Santa Cruz Biotechnology, with epitope mapping amino acids 672-714 of Aβ, i.e., Aβ 1–42), diluted 1:100 in poly-L-ornithine (diluted in 50 mM NaHCO_3_ pH 9.6), were used to coat wells, which were then incubated overnight at 4 °C. After washes, 200-μL/well of bovine serum albumin (BSA) 1% were added to each well and incubated for 1 h at 37 °C to block non-specific sites. Colonic samples were added to each well (10 μg/100 μL) and incubated at 25 °C for 2 h. As primary antibody, 75 μL/well of a mouse monoclonal antibody to α-syn (SC-12767, Santa Cruz Biotechnology, with epitope mapping the carboxy-terminus of Aβ), diluted 1:200 in PBS-BSA-Triton, were employed and incubated at 37 °C for 2 h. Then, 100 μL/well of an anti-mouse-horseradish peroxidase (HRP) antibody, diluted 1:2000 in PBS-BSA-Triton, were used as detection antibody, and incubated at 37 °C for 1.5 h [49]. Next, 100 µL of the chromogenic substrate (3,3′,5,5′-tetramethylbenzidine, TMB, Thermo Scientific) were added to each well. The absorbance was read at 450 nm after the addition of 100 μL of the stop solution (0.4 N HCl) in each well. All measurements were made in duplicate to achieve a minimal inter-assay variability. A recombinant human α-syn solution at different concentrations, diluted in PBS, was used to build the standard curve for ELISA assay [49,50,51,53].

Colonic oligomeric α-syn: Colonic oligomeric α-syn was evaluated by an immunoenzymatic assay, as described previously [47,50]. A mouse monoclonal antibody to α-syn (SC-12767, Santa Cruz Biotechnology) was used to pre-coat wells, and it was left in incubation overnight at room temperature. BSA 1% (200 μL/well) was added for 1 h at 37 °C. The colonic tissue (0.4 μg/100 μL) was introduced into each well and incubated for 2 h at 25 °C. To detect α-syn oligomers, an α-syn biotinylated antibody (that binds human α-syn on the amino acid residues 121–125) (75 μL/well) was employed as primary antibody. A streptavidin HRP conjugate antibody (1:1000, GE Healthcare) was used as detection antibody (100 μL/well). TMB was added to each well (100 μL/well) and colouring was monitored. Then, stop solution (100 μL/well) was added to block the colorimetric reaction. The absorbance was read at 450 nm.

The levels of the interaction oligomeric α-syn in the samples was quantified through a standard curve that was built using different concentrations of recombinant human oligomeric α-syn (Human Alpha Synuclein oligomer ELISA kit, MBS730762, MyBioSource) [49].

Colonic total Aβ: The amount of Aβ in colonic tissue was determined through immuno-enzymatic assay, as described previously [48,49]. A specific rabbit polyclonal antibody to Aβ (SC-9129, Santa Cruz Biotechnology), diluted 1:100 in poli-L-ornithine, was added (60 μL) to each well and maintained overnight at 4 °C. To block aspecific sites, BSA 1% (200 μL/well) was added for 2 h at 37 °C. The colonic tissue (0.25 μg/100 μL) was introduced into each well and incubated at 25 °C for 1 h. A goat polyclonal antibody to Aβ (SC-5399, Santa Cruz Biotechnology) (75 μL/well) was employed and incubated for 1,5 h at 25 °C; then, a donkey anti-goat-HRP antibody (Santa Cruz Biotechnology), diluted 1:2500 in PBS-BSA-Triton, was used against the primary antibody and incubated at 37 °C for 1 h [49]. Conclusively, the wells were incubated with TMB (100 μL/well). After adding the stop solution (100 μL/well), the absorbance was read at 450 nm [49,54].

Faecal total Aβ: Aβ levels in the stools were measured by an ELISA kit (KMB3441, Invitrogen), as previously described [54]. For this purpose, faecal samples (30 mg), stored previously at −80 °C, were weighed, thawed and homogenised in 0.4 mL of 5 M guanidine-HCl/50 mM Tris (pH 8.0 at room temperature) for 3−4 h. Stools were diluted ten-fold with cold PBS with 1× protease inhibitor cocktail (Sigma), centrifuged at 16,000× *g* for 20 min at 4 °C, and then the supernatants were transferred into clean microcentrifuge tubes and kept on ice. Subsequently, a protease inhibitor cocktail with a serine protease inhibitor 1 mM (AEBSF, Sigma) was added since serine proteases can rapidly degrade Aβ peptides. Aliquots (100 μL) of supernatants were then used for the assay. Faecal Aβ levels were expressed as picogram per milligram of feces.

Colonic total tau: The quantification of total tau in colonic tissue was assessed by an immuno-enzymatic assay, as described previously [48,49]. A specific mouse monoclonal antibody to tau (SC-32274, Santa Cruz Biotechnology, with epitope mapping the carboxyl-terminus of tau protein), diluted 1:100 in poly-L-ornithine, was used (60 μL/well) to pre-coat the plate and incubated overnight at 4 °C. BSA 1% (200 μL/well) was added for 1 h at 37 °C, and later on, the colonic tissue (2 μg/100 μL) was incubated for 2 h at 25 °C. A rabbit polyclonal antibody to tau (SC-5587, Santa Cruz Biotechnology) (75 μL/well), diluted 1:250 in PBS-BSA-Triton, was incubated for 2 h at 37 °C. Successively, a goat anti-rabbit-HRP antibody (Invitrogen), diluted 1:2000 in PBS-BSA-Triton, was incubated for 1.5 h. The TMB was added to each well (100 μL/well) and the absorbance was read at 450 nm after adding stop solution (100 μL/well) [49,55].

Colonic p-tau: p-tau levels in colonic tissue were measured through an immuno-enzymatic assay, as described previously [49]. A specific antibody to tau (SC-32274, Santa Cruz Biotechnology), diluted 1:100 in poly-L-ornithine, was used (60 μL/well) to pre-coat the plate and left overnight at 4 °C. BSA 1% (200 μL/well) was added for 2 h at 37 °C. The colonic tissue (1 μg/100 μL) was incubated for 2 h at 25 °C. A polyclonal antibody to tau (70R-32555, Fitzgerald, detecting endogenous levels of tau only when phosphorylated at Thr181) (75 μL/well), diluted 1:5000 in PBS-BSA-Triton, was incubated for 1.5 h at 37 °C. Then, an HRP antibody (Invitrogen), diluted 1:2000 in PBS-BSA-Triton, was incubated for 1.5 h. After the incubation with TMB (100 μL/well), the absorbance was measured at 450 nm after adding the stop solution (100 μL/well) [49].

Colonic α-syn-Aβ heterocomplexes: To quantify the interactions of α-syn with Aβ in colonic tissue, a “home-made” sandwich ELISA system was developed [49,56].

The levels of the interaction between α-syn and Aβ in the samples was quantified through a standard curve [49], which was built using different concentrations of recombinant human α-syn and recombinant human Aβ. The solution was prepared by incubating in parafilm-sealed tubes 1 mg of each protein, in 2 mM SDS, and maintaining it at 37 °C for 36 h in an “Eppendorf Thermomixer” with continuous mixing (500 rpm) [49,57]. All measurements were performed in duplicate to reduce inter-assay variability. Sixty microlitres/well of a rabbit polyclonal antibody to Aβ (SC-9129, Santa Cruz Biotechnology), diluted 1:100 in poly-L-ornithine, were used to pre-coat wells and incubated overnight at room temperature. The colonic tissue (1 μg/100 μL) was added to each well and incubated for 2 h at 25 °C. Two hundred microlitres of BSA 1% were added to each well for 20 min at 37 °C, to block aspecific sites. Seventy-five microlitres of mouse monoclonal anti-α-syn antibody (SC-12767, Santa Cruz Biotechnology), diluted 1:200 in Milk 5%, were employed and incubated at 37 °C for 2 h. Subsequently, 100 µL of goat anti-mouse-HRP antibody (Santa Cruz), diluted 1:2000 in Milk 5%, were incubated for 1.5 h at 37 °C [49]. Then, 100 µL/well of TMB were added. Absorbance was evaluated at 450 nm after adding 100 μL/well of stop solution.

Colonic α-syn-tau heterocomplexes: To detect quantitatively the interactions of α-syn with tau in colonic tissue, a “home-made” sandwich ELISA was developed [49,50,51,56]. The levels of the interaction between α-syn and tau in tissue samples was quantified through a standard curve [49], which was built using different concentrations of recombinant human α-syn and recombinant human tau. The solution was prepared by incubating in parafilm-sealed tubes 1 mg of each protein, diluted in 2 mM SDS, and maintaining it at 37 °C for 1 h in an Eppendorf Thermomixer with continuous mixing (500 rpm) [49,50,51]. All measurements were performed in duplicate to lower inter-assay variability. A pre-coating of the ELISA plate was carried out using a goat polyclonal α/β-syn antibody (SC-7012, Santa Cruz Biotechnology), diluted 1:100 in poly-L-ornithine, left overnight at room temperature. After incubation of the colonic samples (1 μg/100 μL) in each well for 2 h at 25 °C, BSA 1% (200 μL/well) was added for 20 min at 37 °C to block aspecific sites. As primary antibody, a rabbit polyclonal anti-tau antibody (SC-5587, Santa Cruz Biotechnology), diluted 1:200, was employed for capturing at 37 °C for 2 h. Subsequently, a goat anti-rabbit-HRP antibody was used, as detection antibody, at 37 °C for 1.5 h [49]. One hundred microlitres/well of TMB were added in each well and the colour was allowed to develop for 30 min at room temperature. Absorbance was measured at 450 nm after adding 100 μL/well of stop solution.

### 4.9. Evaluation of Tissues IL-1β Levels

Interleukin (IL)-1β levels in colonic tissues were measured by ELISA kit (R&D system), as described previously [58,59]. For this purpose, colonic tissue samples, stored previously at −80 °C, were weighed, thawed and homogenised in 0.4 mL of PBS, pH 7.2/20 mg of tissue at 4 °C, and centrifuged at 10,000× *g* for 5 min. Aliquots (100 μL) of supernatants were then used for the assay. Tissue IL-1β levels were expressed as picogram per millilitre.

### 4.10. Evaluation of Tissue Citrate Synthase Activity

Frozen colonic tissues were homogenised on ice with an ultra-turrax homogeniser (IKA-Werke GmbH & Co., Germany) in a cold buffer (composition: sucrose 250 mM, Tris 5 mM, EGTA 1 mM, Triton X-100 0.02%, pH 7.4). Then, homogenates were centrifuged at 12,000× *g* for 15 min at 4 °C (EuroClone, Speed Master 14 R centrifuge, Italy). The supernatant was used for determination of the citrate synthase activity, and the protein concentration in the supernatant was determined spectrophotometrically by Bradford assay (Bio-Rad, USA), using a microplate reader (EnSpire, PerkinElmer, USA). Then, proteins were diluted in Tris-buffer 100 mM (pH 8.2) containing 5,5′-dithiobis-(2-nitrobenzoic) acid (DTNB, 100 µM) and acetyl-coenzyme A (100 µM). The assay was performed in 96-well plates (1 µg of proteins per well) and the reaction was initiated by addition of oxalacetate 500 µM. The absorption of the reaction product was measured spectrophotometrically at 30 °C and 412 nm every 30 s for 15 min. Citrate synthase activity was determined by comparing the activity in the samples to that of known concentrations of the isolated enzyme (Sigma-Aldrich, St. Louis, MI, USA). Citrate synthase activity was expressed in mU/µg protein. Data were analysed by a computer fitting procedure (software: GraphPad Prism 5.0).

### 4.11. Western Blot Analysis of NLRP3, ASC and Caspase-1 Expression

The colonic tissues were weighed and homogenised in lysis buffer, using a polytron homogeniser, as described by Richter et al. [60]. Proteins were quantified with the Bradford assay. Proteins (30 μg) were separated onto a pre-cast 4-20% polyacrylamide gel (Mini-PROTEAN^®^ TGX gel, Biorad) and transferred to PVDF membranes (Trans-Blot^®^ TurboTM PVDF Transfer packs, Biorad). Membranes were blocked with 3% BSA diluted in Tris-buffered saline (TBS, 20 mM Tris-HCl, pH 7.5, 150 mM NaCl) with 0.1% Tween 20. Primary antibodies against β-actin (monoclonal, diluted 1:5000, A5316, Sigma Aldrich), nucleotide-binding oligomerisation domain leucine rich repeat and pyrin domain containing protein 3 (NLRP3) (polyclonal, diluted 1:1000, ab214185, Abcam), apoptosis-associated speck-like protein containing a caspase recruitment domain (ASC) (monoclonal, diluted 1:1000, D2W8U, Cell Signaling) and caspase-1 (polyclonal, diluted 1:1000, ab1872, Abcam) were used. Secondary antibodies were obtained from Abcam (anti-mouse ab97040, Abcam, and anti-rabbit ab6721, Abcam). Protein bands were detected with ECL reagents (Clarity™ Western ECL Blotting Substrate, Biorad). Densitometry was performed by ImageJ software.

### 4.12. Histological Evaluation of Eosinophils

Sections from formalin-fixed full-thickness colonic samples were processed for routine (haematoxylin and eosin) and histochemical staining (0.1% toluidine blue in 30% ethanol for 15 min) in order to detect eosinophils, which appeared as deep violet cells. The density of eosinophils was assessed in the tunica mucosa/submucosa. Cells were counted in three different sections from each mouse, and at least 20 randomly selected microscopic fields were examined in each section (objective, 40×). Values obtained from all the examined fields for each rat were averaged and expressed as cell number per square millimetre of tunica mucosa/submucosa areas, which were estimated by the Image Analysis System “L.A.S. software v.4.” These values were then used to calculate mean values for each experimental group.

### 4.13. Immunohistochemistry of Claudin-1

Sections (epithelial cells) were incubated overnight at 4 °C with the primary anti-claudin-1 antibody. Sections were then exposed to appropriate biotinylated immunoglobulins, peroxidase-labelled streptavidin complex and 3.3′-diaminobenzidine tetrahydrochloride, counterstained and examined by a Leica DMRB light microscope equipped with a DFC480 digital camera (Leica Microsystems, Cambridge, UK). For each group, representative photomicrographs were shot and analysed quantitatively using the Image Analysis System “L.A.S. software version 4.5” (Leica Microsystems, Cambridge, UK). Two blind investigators (C.I. and C.S.) carried out cell-counting independently and assessed the colorimetric threshold values to detect antigen expression levels. Immunostaining expression was calculated as ratio between area of the stained fields and the total tissue area examined (percentage positive pixels (PPP)), as previously reported [58,61]. Data obtained from all the examined fields for each rat were averaged and used to calculate mean values ± SEM for each experimental group, which were plotted in graphs.

### 4.14. In Vitro Assays on NLRP3 Inflammasome

Wilde-type (WT) and ASC knock out (ASC^-/-^) human monocytic cell lines (THP-1) were donated by Prof. Veit Hornung (Ludwig Maximilian University of Munich) and cultured in RPMI 1640 media (Sigma) supplemented with 10% FBS (PAA Laboratories), 100 units/mL penicillin, and 100 µg/mL streptomycin (Sigma). Cells were plated in 24-well plates at a density of 5 × 10^5^ cells/well and treated with phorbol 12-myristate 13-acetate (PMA, 0.5 µM). After 3 h, the medium was removed, fresh media was added, and cells were incubated overnight (37 °C, 5% CO_2_).

In the first series of experiments, cells were lipopolysaccharide (LPS)-primed (1 µg/mL, 4 h) to induce pro-IL-1β expression before treatment with nigericin (a standard NLRP3 inflammasome activator, 10 µM, 1 h) or Aβ (5, 10 and 15 µM, 6 h), as described by [24,62,63].

In the second series of experiments, LPS-primed (1 µg/mL, 4 h) cells were treated for 15 min with vehicle (0.5% dimethyl sulfoxide, DMSO) or caspase-1 inhibitor (YVAD, 100 µM) before the addition of nigericin (10 µM, 1 h) or Aβ (10 µM, 6 h), as described by [24].

In the third series of experiments, LPS-primed (1 µg/mL, 6 h) ASC^-/-^ THP-1 cells were treated with nigericin (standard NLRP3 inflammasome activator, 10 µM, 1 h) or Aβ (10 µM, 6 h).

WT and ASC^-/-^ THP-1 treated with nigericin or Aβ in the presence or absence of YVAD, respectively, were incubated for 1 h or 6 h, respectively, before the collection of supernatants and the lysis of cells for analysis of IL-1β processing and release. IL-1β in cell supernatants was quantified by an ELISA kit (R&D Systems), following the protocols provided by the manufacturer. IL-1β concentration was expressed as picogram per millilitre.

#### 4.14.1. Cell Death Measurement

Cell death was measured using quantitative assessment of lactate dehydrogenase (LDH) levels in the medium. CytoTox 96^®^ Non-Radioactive Cytotoxicity Assay (G1780, Promega) was used in accordance with manufacturer instructions. Plates were read at 490 nm and results are shown as percentage of LDH release relative to the total cells lysed.

#### 4.14.2. ASC Speck Detection and Quantification

Cells were plated as described above on glass coverslips. THP-1 cells were LPS-primed (1 µg/mL, 4 h) and treated with nigericin (10 µM, 1 h) or Aβ (10 µM, 6 h). Cells were then fixed with 4% paraformaldehyde and 4% sucrose in PBS for 30 min. The cells were permeabilised with 0.1% Triton X-100 and then quenched with 0.25% ammonium chloride. A blocking step for 1 h using 5% BSA, and 5% donkey serum (block solution) was used before incubation with the rabbit anti-ASC (1:500). Coverslips were then washed in PBS. ASC antibodies were detected by incubation with Alexa Fluor 594 conjugated donkey anti-rabbit antibody (1:1000) in blocking solution for 1 h. The coverslips were washed again with PBS and finally in distilled water before being dried and mounted onto a glass slide using ProLong Gold mounting medium containing 4′,6-diamidino-2-phenylindole dihydrochloride (DAPI) (Invitrogen). Images were taken with an Olympus BX51 upright microscope using a 20×/0.50 Plan Fln objective and captured using a Coolsnap EZ camera (Photometrics) through MetaVue Software (Molecular Devices). To quantify the extent of speck formation, the percentage of cells that contained an ASC speck was counted. Cells from 10 different fields (average of 650 cells/field) were counted for each of the different experiments (*n* = 3). Images were analysed using ImageJ (rsb.info.nih.gov). Data are expressed as the percentage of ASC specks per number of cells per field.

#### 4.14.3. Flow Cytometry Analysis of Mitochondrial Potential

Mitochondrial membrane potential variations are related to apoptotic process, necrotic cell death and caspase-independent cell death. Depolarisation of the inner mitochondrial membrane potential is a reliable indicator of mitochondrial dysfunction and cellular health. Mitochondrial potential was analysed by flow cytometry using the Muse™ MitoPotential Assay Kit from EMD Millipore Bioscience. Mitochondrial potential was evaluated according to the manufacturer’s protocol. Briefly, THP-1 cells were seeded onto 24-well plates at a density of 5 × 10^4^ per well. After 24 h, cells were treated for 6 h with Aβ 10 µM and for 4 h with LPS 1 µg/mL followed by nigericin 2-h treatment at 10 µM, or with no-treatment. After incubation, 1 × 10^4^ cells were resuspended in the 1× Assay Buffer provided. Next, 95 µL of Muse™ MitoPotential working solution were added to 100 μL of cell suspension and incubated at 37 °C for 20 min protected from light. Then, 5 µL of Muse™ 7-AAD working solution were added and incubated at 37 °C for 5 min; cells were analysed by the Muse™ Cell Analyzer.

### 4.15. Drugs and Reagents

Aβ was purchased by Biochem (Germany). Atropine sulphate, SP, guanethidine monosulphate, nigericin, Ac-YVAD-cmk, bacterial LPS (*Escherichia coli* 026:B6) and DMSO, sucrose, Tris, EGTA, Triton X-100, Tris-buffer, DTNB, acetil-coenzyme A, oxaloacetate and anti-β-actin-HRP (A3854) were purchased from Sigma Chemicals Co. (St. Louis, MO, USA). Foetal bovine serum (FBS) was obtained from PAA Laboratories. TTX, GR159897, SB218795, L-NAME were obtained from Tocris (Bristol, UK). Anti-IL-1β (anti-human) was purchased by R&D, rabbit anti-ASC (SC-22514-R) by Santa Cruz. Secondary antibody HRP conjugates were from DAKO. Alexa Fluor 594-conjugated donkey anti-rabbit antibody (A-21207) was purchased by Invitrogen.

### 4.16. Statistical Analysis

The results are presented as mean ± S.E.M. unless otherwise stated. The significance of differences was evaluated by two-way analysis of variance (ANOVA) followed by post hoc analysis with the Fisher LSD test (for paired data), one-way ANOVA followed by post hoc analysis with Bonferroni post hoc test (for paired data) or Student t test (for unpaired data) where appropriate. *p* values < 0.05 were considered significantly different. All statistical procedures were performed by commercial software (GraphPad Prism, version 7.0 from GraphPad Software Inc., San Diego, CA, USA).

## 5. Conclusions

The present study provides evidence that, in the SAMP8 AD model, cognitive dysfunctions are associated with enteric AD-related protein accumulation and their heterocomplexes, colonic inflammation, mitochondrial dysfunction, altered IEB and impaired excitatory cholinergic and tachykininergic neurotransmission, which may all contribute to bowel motor dysfunctions since the earliest stages of the disease, before the full development of brain pathology. In this context, inflammasome activation might represent the crossroad between the shaping of enteric neurogenic/inflammatory responses and the onset of bowel motor alterations.

It must be acknowledged, however, that our results do not allow to establish clearly whether the intestinal changes contribute to brain pathology, or whether they occur rather as a consequence of the initiation of central neurodegeneration. In this regard, several lines of evidence support the contention that alterations of the enteric bacteria-neuro-immune network, besides determining intestinal dysfunctions, may contribute also to the pathogenesis of AD [26,64,65]. In particular, changes in gut microbiota composition can promote the pathological accumulation of enteric Aβ protein. Enteric Aβ, regarded also as a prion-like proteinaceous nucleating particle, could then move through myenteric neurons and spread to the CNS, via the neuronal gut–brain axis, contributing directly to the pathogenesis of AD [66,67]. In parallel, the enteric Aβ-protein deposition could shape enteric and peripheral neurogenic/inflammatory responses (i.e., activation of NLRP3 inflammasome) and contribute to both bowel motor dysfunctions and neuroinflammation/neurodegeneration in the CNS, via immune gut–brain ascending pathways [5]. However, whether the Aβ-induced NLRP3 activation, besides shaping immune/inflammatory responses, contributes also to alter the enteric neuronal pathways, or whether both events occur concomitantly, remains to be clarified. In addition, whether the Aβ prion-like protein spreads to the CNS, or whether, through NLRP3 activation, it triggers peripheral and central immune/inflammatory responses, via immune–gut–brain pathways, contributing to brain pathology, remains unclear and deserve further investigations. Moreover, given the relevance of the relationship among gut microbiota, diet and Aβ accumulation, the characterisation of enteric bacteria alterations in SAMP8 mice since the earliest stages of disease, their role in promoting Aβ-protein accumulation, and the impact of diet (i.e., Mediterranean diet, including polyunsaturated fatty acids proteins, vitamins, polyphenols and fibres) in counteracting enteric Aβ-protein accumulation, inflammation, bowel motor symptoms and CNS pathology, remains to be clarified and could represent the logical continuation in this research topic.

Another gap in our study concerns whether the enteric neurogenic/inflammatory alterations in SAMP8 animals could depend on their accelerated ageing, rather than AD-related cognitive impairment. In this regard, we provide evidence that cognitive dysfunctions in SAMP8 animals are associated with enteric AD-related protein accumulation and their heterocomplexes, colonic inflammation, mitochondrial dysfunction, altered IEB and impaired excitatory cholinergic and tachykininergic neurotransmission, which could contribute to bowel motor dysfunctions since the earliest stages of the disease. In addition, in the in vitro experiments, we observed that AD-related proteins, with particular regard for Aβ, promoted inflammasome activation and mitochondrial depolarisation, thus suggesting that the enteric AD-related protein deposition could trigger immune/inflammatory responses and mitochondrial dysfunctions that, in turn, could contribute to bowel dysmotility. Therefore, it is conceivable that the enteric changes observed in SAMP8 mice depend on AD-related protein accumulation in intestinal tissues. However, future investigations in SAMP8 mice at different ages, aimed at evaluating the occurrence of pathological AD-related protein deposition in colonic tissues and bowel dysfunctions, could allow establishing a temporal relationship between intestinal alterations and AD progression.

Overall, these results can provide a basis for better understanding of the mechanisms underlying bowel motor disturbances in AD, thus paving the way to the identification of novel pharmacological approaches to the management of intestinal symptoms associated with AD.

## Supplementary Materials

The following are available online at https://www.mdpi.com/1422-0067/21/10/3523/s1, Figure S1: Immunofluorescence of apoptosis-associated speck-like protein containing a caspase recruitment domain (ASC) ASC Speck Detection. Immunofluorescence images of apoptosis-associated speck-like protein containing a caspase recruitment domain (ASC) expression in lipopolysaccharide (LPS)-primed (1g/mL, 4 h) human monocytic cell lines (THP-1) treated with nigericin (Nig, 10 μM, 1 h) or amyloid β (Aβ, 10 μM, 6 h). Scale bar = 50 μM.

## Abbreviations

AAMI	Age-associated memory impairment
Aβ	Amyloid β1-42
AD	Alzheimer’s disease
AβPP	Aβ protein precursor
ASC	Apoptosis-associated speck-like protein containing a caspase recruitment domain
α-syn	α-synuclein
BSA	Bovine serum albumin
CCh	Carbachol
ChAT	Choline acetyltransferase
CNS	Central nervous system
DAPI	4′,6-diamidino-2-phenylindole
DMSO	Dimethyl sulfoxyde
DTNB	5,5′-dithiobis-(2-nitrobenzoic) acid
ELISA	Enzyme-linked immunosorbent assay
ES	Electrical stimulation
HRP	Horseradish peroxidase
IEB	Intestinal epithelial barrier
IL-1β	Interleukin-1beta
LDH	Lactate dehydrogenase
L-NAME	Nω-nitro- L-arginine methylester
LPS	Lipopolysaccharide
MCI	Mild cognitive impairment
MWM	Morris water maze test
NK1	Neurokinin 1
NLRP3	Nucleotide-binding oligomerisation domain leucine rich repeat and pyrin domain containing protein 3
nNOS	Neuronal nitric oxide synthase
PBS	Phosphate buffered saline
PMA	Phorbol 12-myristate 13-acetate
p-tau	Phosphorylated tau
SAMP8	Senescence-accelerated mouse prone 8
SAMR1	Senescence-accelerated mouse-resistant 1
SDS	Sodium dodecyl sulphate
SP	Substance P
TMB	3,3′,5,5′-tetramethylbenzidine
TTX	Tetrodotoxin
