# Supplementary material for: Prodromal Intestinal Events in Alzheimer’s Disease (AD): Colonic Dysmotility and Inflammation Are Associated with Enteric AD-Related Protein Deposition
Source: Int J Mol Sci. 2020 May 15;21(10):3523. doi: 10.3390/ijms21103523 (PMC7278916; doi:10.3390/ijms21103523)
Supplement: Supplementary file 1 [file ijms-21-03523-s001.pdf]

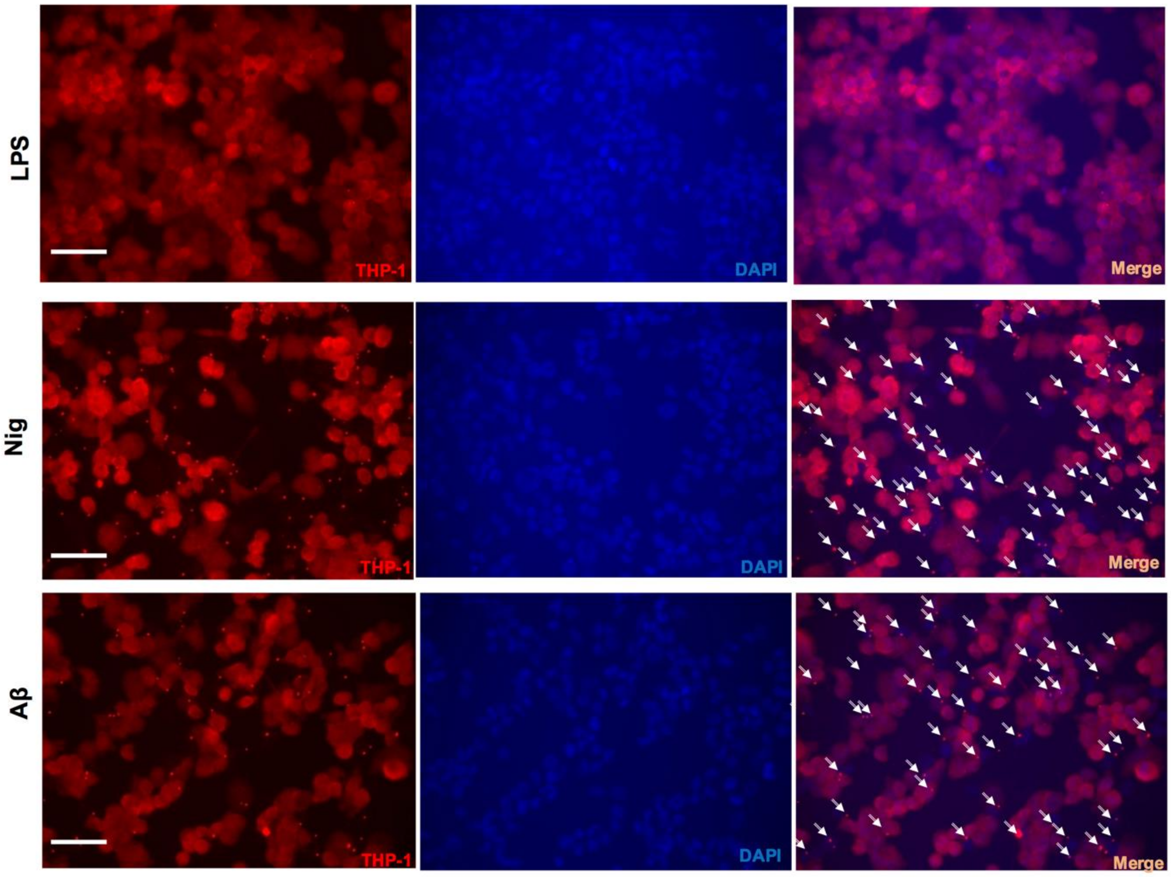

Supplemental figure 1. Immunofluorescence of apoptosis-associated speck-like protein containing a caspase recruitment domain (ASC) ASC Speck Detection. Immunofluorescence images of apoptosis-associated speck-like protein containing a caspase recruitment domain (ASC) expression in lipopolysaccharide (LPS)-primed (1g/mL, 4 h) human monocytic cell lines (THP-1) treated with nigericin (Nig, 10 μM, 1 h) or amyloid β (Aβ, 10 μM, 6 h). Scale bar = 50 μm.
